# Free Thyroxine Levels are Associated with Cold Induced Thermogenesis in Healthy Euthyroid Individuals

**DOI:** 10.3389/fendo.2021.666595

**Published:** 2021-06-14

**Authors:** Claudia Irene Maushart, Jaël Rut Senn, Rahel Catherina Loeliger, Marius E. Kraenzlin, Julian Müller, Anton S. Becker, Miroslav Balaz, Christian Wolfrum, Irene A. Burger, Matthias Johannes Betz

**Affiliations:** ^1^ Department of Endocrinology, Diabetes and Metabolism, University Hospital Basel, University of Basel, Basel, Switzerland; ^2^ SpezialLABOR Hormone - Knochenstoffwechsel, University of Basel, Basel, Switzerland; ^3^ Department of Radiology, University Hospital Zurich, Zurich, Switzerland; ^4^ Health Sciences and Technology, Institute of Food, Nutrition and Health, ETH Zurich, Schwerzenbach, Switzerland

**Keywords:** brown adipose tissue, cold adaptation, cold induced thermogenesis, energy expenditure, thyroid hormone, thyroxine

## Abstract

Thyroid hormone (TH) is an important regulator of mammalian metabolism and facilitates cold induced thermogenesis (CIT) in brown adipose tissue (BAT). Profound hypothyroidism or hyperthyroidism lead to alterations in BAT function and CIT. In euthyroid humans the inter-individual variation of thyroid hormones is relatively large. Therefore, we investigated whether levels of free thyroxine (T4) or free triiodothyronine (T3) are positively associated with CIT in euthyroid individuals. We performed an observational study in 79 healthy, euthyroid volunteers (mean age 25.6 years, mean BMI 23.0 kg · m^-2^). Resting energy expenditure (REE) was measured by indirect calorimetry during warm conditions (EE_warm_) and after a mild cold stimulus of two hours (EE_cold_). CIT was calculated as the difference between EE_cold_ and EE_warm_. BAT activity was assessed by ^18^F-FDG-PET after a mild cold stimulus in a subset of 26 participants. EE_cold_ and CIT were significantly related to levels of free T4 (R^2^ = 0.11, p=0.0025 and R^2^ = 0.13, p=0.0011, respectively) but not to free T3 and TSH. Cold induced BAT activity was also associated with levels of free T4 (R^2^ = 0.21, p=0.018). CIT was approximately fourfold higher in participants in the highest tertile of free T4 as compared to the lowest tertile. Additionally, free T4 was weakly, albeit significantly associated with outdoor temperature seven days prior to the respective study visit (R^2^ = 0.06, p=0.037). These finding suggests that variations in thyroid hormone levels within the euthyroid range are related to the capability to adapt to cool temperatures and affect energy balance.

## Introduction

Thyroid hormone (TH) is an important regulator of mammalian metabolism and resting energy expenditure (REE) (1). Overt hyperthyroidism increases REE and food intake (2), and stimulates gluconeogenesis (3) as well as lipolysis (4) while overt hypothyroidism has the opposite effect (5–7). However, the consequences of subtle changes in thyroid hormone levels on metabolism are less certain. Several cross-sectional studies suggest that variations of free thyroxine (free T4) and free triiodothyronine (free T3) within the reference range are associated with metabolic health (8–11). In mammals, TH is crucial for the maintenance of body core temperature by positively regulating thermogenesis (12). TH affects virtually all metabolically active tissues and among others Brown adipose tissue (BAT) is an important target of TH action (13). BAT is a thermogenic tissue and is activated by the sympathetic nervous system in response to mild cold exposure. The resulting increase in REE is called “cold induced thermogenesis” (CIT) (13). Brown adipocytes contain a high amount of mitochondria which harbor uncoupling protein 1 (UCP1) in the inner mitochondrial membrane. UCP1 is exclusive to brown adipocytes and can convert chemical energy directly into heat by short-circuiting oxidative phosphorylation (14). Research in both rodents (15) and humans (16) indicates favorable effects of active BAT on metabolism, such as a reduction in blood glucose and triglycerides and lipoproteins suggesting that more active BAT could contribute to a metabolically healthy phenotype.

Recently, we demonstrated that CIT is reduced in hypothyroid individuals and can be restored by sufficient thyroid hormone replacement (17). In the present study, we investigated whether levels of thyroid hormones within the reference range in healthy, euthyroid individuals are connected to EE at warm and cold temperatures.

## Materials and Methods

### Subjects

We collected data of healthy volunteers from a prospective observational study [clinicaltrials.gov ID: NCT02682706 (18)] and the screening data from two interventional trials [NCT03189511 (19) and NCT03269747]. All participants were recruited *via* local advertisement at the endocrine outpatient clinic at the University Hospital Basel. Inclusion criteria were as follows: age 18 to 40 years, female and male persons with a BMI of 17.5 to 27 kg/m^2^ for NCT02682706. Male persons with a BMI of 17.5 to 27 kg/m^2^ for NCT03189511 and NCT03269747. We excluded participants with chronic heart failure, liver cirrhosis, kidney failure, active cancer, thyroid hormone disorders or intake of the following medication: Non-steroidal anti-inflammatory drugs (NSAID), glucocorticoids, diuretics, antihypertensives, fibrates or statins, metformin. From March 2016 to February 2019 we included a total of 79 healthy participants.

The ethical review board of Northwest and Central Switzerland (EKNZ) approved the studies and all participants provided written informed consent.

### Energy Expenditure and Cold Induced Thermogenesis

Resting energy expenditure (REE) was measured for 30 minutes by indirect calorimetry using a ventilated canopy calorimeter (Quark RMR, Cosmed, Rome, Italy). Participants fasted for at least 6 hours prior to the study visit and were asked to refrain from intensive physical activity 24 hours before the scheduled study visit. We measured EE during warm conditions (EE_warm_) and after a standardized, mild cold stimulus (EE_cold_). All visits took place in an air-conditioned study room at a controlled ambient temperature of 24°C year round. For determination of EE_warm_ participants were placed in a hospital bed in a supine position and were covered with a fleece blanket. After the first measurement, the blanket was removed and the patients were asked to wear only a t-shirt and shorts. Participants were additionally exposed to mild cold using a water circulated cooling system (Hilotherm clinic, Hilotherm GmbH, Germany) around the patient’s waist. The water temperature was continuously reduced by 1°C every two minutes from 25°C to a minimum of 10°C. During the cooling participants were asked repeatedly if they experienced cold or noticed shivering. In case of shivering they were covered with a blanket for 5 minutes and the water temperature was raised by 2°C until the shivering stopped. The total cooling time was 120 minutes. During the last 30 minutes of the cooling the measurement of EE_cold_ was performed. Cold induced thermogenesis (CIT) was calculated as the difference between EE_cold_ and EE_warm_. Relative CIT was calculated as the percentage of EE_warm_ in the respective subject in order to correct for variations in size.

### Measurements of Skin and Tympanic Temperatures

During the experiment we continuously measured the skin temperature every 60 seconds with self-contained temperature probes (iButton DS1922L, Maxim Integrated Products, Inc, San Jose, CA) at the following eight defined body locations: Supraclavicular region (right and left), parasternal at the level of the 2nd intercostal space (right and left), umbilicus, mid-thigh (right and left), middle of the lower arm palmar side, finger tip of the 3rd finger of the non-dominant hand, and middle of the lower left leg, back of the left foot. The temperature data of every location during the last ten minutes of warm and cold phase, respectively, were averaged.

The tympanic temperature was measured with infrared tympanometry (Braun, ThermoScan PRO 6000, Marlborough, MA) before and after cold exposure.

### ^18^F-FDG-PET/CT and ^18^F-FDG-PET/MR Measurements

In a subset of participants we analyzed BAT activity by ^18^F-FDG-PET which was performed after controlled cold exposure in 17 and 16 participants in the context of two clinical trials [trial 1: NCT03189511 (19) and trial 2: NCT03269747]. Specifically, the participants who qualified for the interventional trials continued to ^18^F-FDG-PET. Briefly, participants were exposed to mild cold for two hours using a Hilotherm clinic cooling device as described above. In trial 1 BAT was further stimulated by oral administration of 200 mg of Mirabegron 90 minutes before the onset of cooling (Betmiga, Astellas Pharma, Wallisellen, Switzerland). In both trials, participants received 75 MBq ^18^F-FDG intravenously directly after the cold exposure. PET scans were performed on a SIGNA PET/MR (GE Healthcare, Waukesha, WI, USA; trial 1) or a Siemens Biograph mCT (Siemens Healthineers, Erlangen, Germany; trial 2), respectively. We compared the SUV_mean_ of supraclavicular adipose tissue to the levels of free T4 measured before the stimulation of BAT. In this setting, TH was measured in 10 participants from trial 1 and 16 participants from trial 2.

### Laboratory Analysis

Blood was sampled into serum monovettes (Sarstedt, Germany) at the beginning of the study visit with all subjects fasted for at least six hours. They were also asked to refrain from strenuous exercise during the 24 hours before attending the study. Samples were left standing for 30 minutes and were then centrifuged at 3000xg at 4°C.

TSH and free T4 were measured at the central lab of the University Hospital Basel. For TSH and free T4 electro-chemiluminescence immunoassay (Elecsys, all assays from Roche Diagnostics AG, Rotkreuz, Switzerland) were used. The reference range for TSH was 0.33 – 4.49 mIU · l^-1^. The fT4 levels had a reference range of 11.6-22.0 pM. The reference ranges were established at the central lab of the University Hospital Basel.

Thyroglobulin and free T3 were analysed at a specialized endocrine laboratory (SpezialLABOR, Basel, Switzerland) on a Siemens Immulite 2000 Systems (Siemens Healthcare Diagnostic Products Ltd., Gwynedd, UK). The reference range was 2.8–6.5 pM for free T3 and 1.6 and 59.9 ng · ml^-1^ for thyroglobulin. Reference ranges for these parameters were according to the manufacturer’s package insert.

### Meteorological Data

The Institute for Meteorology, Climatology and Remote Sensing at the University of Basel provided daily mean, maximum and minimum temperatures for all days during the study period. The outdoor temperatures were recorded at an urban meteorological station nearby the University Hospital Basel. Mean daily temperatures were averaged over a period of 7 days prior to the respective study visit.

### Statistical Analysis

Data were analysed using R Version 3.5 (20) and GraphPad Prism Version 9 (GraphPad, La Jolla, CA). Continuous data are given as mean ± SD unless stated otherwise. TSH values were not normally distributed and log-transformed before further analysis. Simple linear regression was performed for measures of EE and TH levels. In order to correct for potential confounding factors linear modeling was performed as follows: All continuous variables were scaled by Z-scaling. A linear model was built in R containing all anthropometric and laboratory values. Thereafter the model was refined stepwise in order to reduce the number of variables to only significant ones. A p-value below 0.05 was considered significant.

## Results

### Baseline Characteristics

All participants were healthy, euthyroid and had a mean age of about 25 years. Due to the study design of the two interventional trials, the majority of subjects was male. Baseline anthropometric data and thyroid hormone values are given in **Table 1**.

**Table 1:** Clinical characteristics at baseline.

	Baseline (n = 79, mean ± SD)
**Age**	25.6 ± 5.4
**Sex (% male)**	87% (69 male, 10 female)
**Weight (kg)**	74.1 ± 9.8
**Height (cm)**	179.1 ± 7.0
**BMI (kg · m^-2^)**	23.0 ± 2.3
**TSH (mU · L^-1^)**	2.0 ± 0.9
**free T4 (pM)**	16.5 ± 2.2
**free T3 (pM)**	5.1 ± 0.69

### Skin and Tympanic Temperature in Response to Cooling

**Figure 1:**
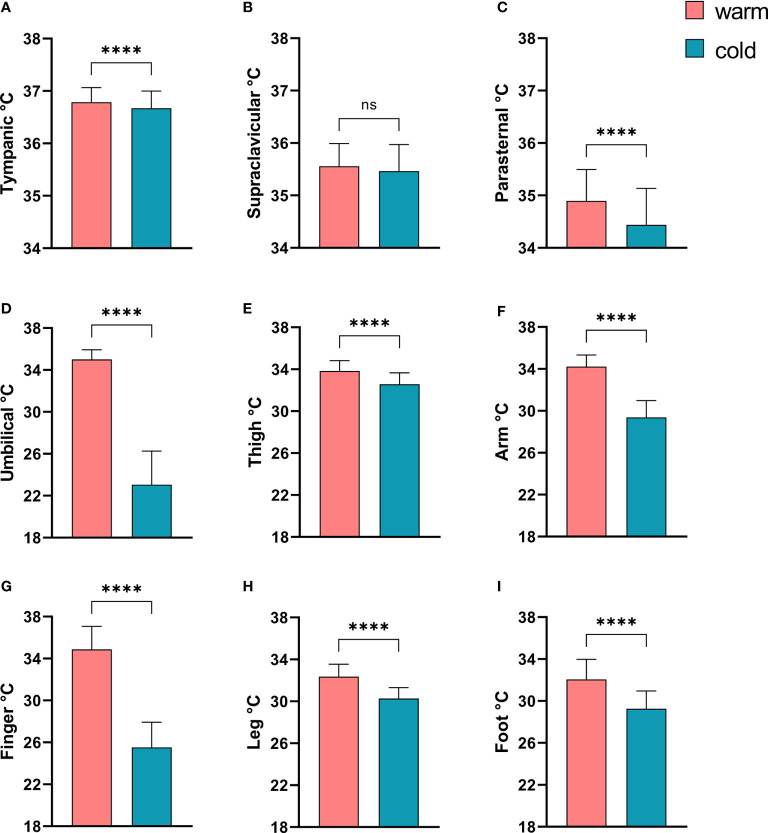
Comparison of tympanic temperature and skin temperatures before and after mild cold exposure. **(A)** Tympanic temperature; Skin temperatures: **(B)** Supraclavicular region; **(C)** Parasternal region; **(D)**, Umbilicus; **(E)** Thigh; **(F)** Non-dominant forearm; **(G)** Middle finger, non dominant hand; **(H)** Left lower leg; **(I)** Left dorsal foot. ****p<0.0001 in Wilcoxon-Signed-Rank Test, ns, p≥0.05.

In order to document a sufficient cold exposure we compared the skin and tympanic temperatures in all subjects before and after cold exposure. The tympanic temperature dropped only minimally, albeit significantly (see **Figure 1A**). With the exception of the supraclavicular region, the skin temperature dropped significantly in all other skin regions. The reduction of skin temperature was most pronounced in the umbilical region as the sensor was intentionally placed below the cooling mattress placed around the patients’ mid-section (see **Figures 1B–I**).

### Association of Resting Energy Expenditure to TSH, Free T4 and Free T3

**Figure 2:**
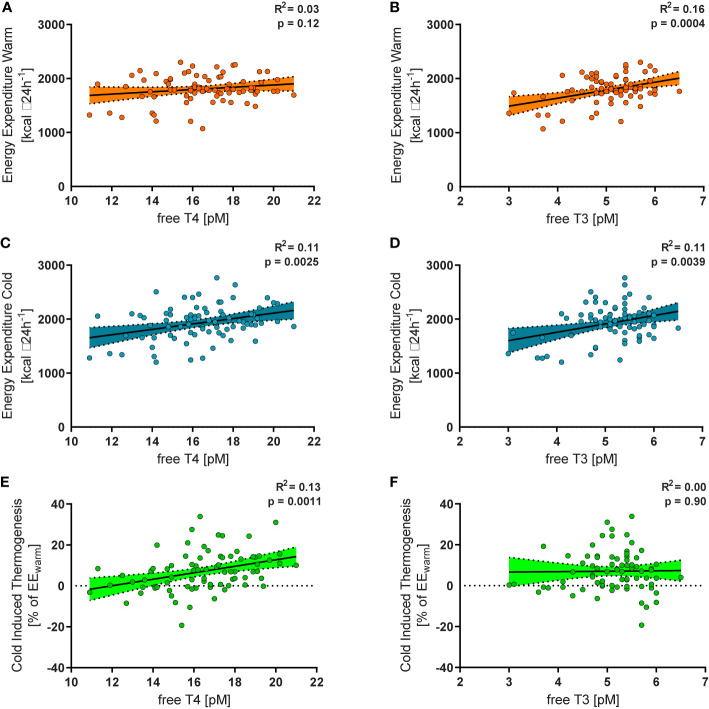
Free T4 within the reference range was not significantly related to energy expenditure during warm conditions (EE_warm_, R^2^ = 0.03, p=0.12, **(A)**, but to energy expenditure during mild cold exposure (EE_cold_, R^2^ = 0.11, p=0.0025, **(B)** and to relative cold induced thermogenesis (CIT, R^2^ = 0.13, p=0.0011, **(C)**. Free T3 was significantly associated with EE_warm_ (R^2^ = 0.16, p=0.0004, **(D)**, EE_cold_ (R^2^ = 0.11, p=0.0039, **(E)**, but not with CIT (R^2^ = 0.00, p=0.90, **(F)**.

In all study subjects, resting energy expenditure (REE) was measured during warm conditions (EE_warm_) and after a mild cold stimulus of two hours (EE_cold_). TSH was not associated with CIT (p=0.37), EE_warm_ (p=0.82) or EE_cold_ (p=0.78). While EE_warm_ was not significantly associated with levels of free T4 (R^2^ = 0.03, p=0.12, **Figure 2A**) it correlated significantly with levels of free T3 (R^2^ = 0.16, p=0.0004, **Figure 2D**). EE_cold_ was weakly, albeit significantly associated with levels of free T4 (R^2^ = 0.11, p=0.0025, **Figure 2B**) and with free T3 (R^2^ = 0.11, p=0.0039, **Figure 2E**).

### Association of Free T4 and Cold Induced Thermogenesis

**Figure 3:**
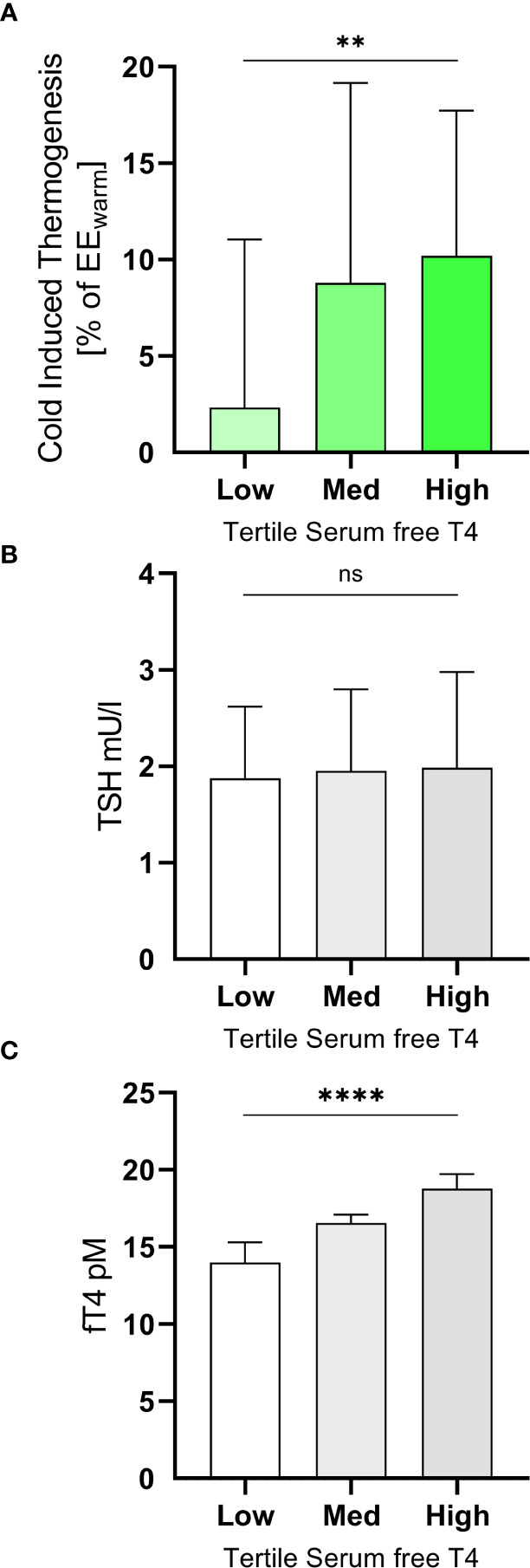
Subjects were grouped into tertiles according their levels of free T4 **(A)**. The mean TSH levels of the three groups did not differ significantly (ANOVA p=0.89, **(B)**. In the highest tertile (free T4 17.5 to 21.0 pM) they had mean CIT of 10.2 ± 7.5% of EE_warm_ while those in the lowest tertile (free T4 10.9 to 15.7 pM) had a mean CIT of 2.3 ± 8.7% (ANOVA p=0.0057, trend p=0.0027, **(C)**. **p<0.01; ****p<0.0001; ns, p≥0.05.

Cold induced thermogenesis (CIT) was calculated as the difference between EE_cold_ and EE_warm_. For a better comparability of the interindividual variation of REE we calculated relative CIT (CIT divided by EE_warm_). Relative CIT was significantly associated with free T4 (R^2^ = 0.13, p=0.0011, **Figure 2C**) but not with free T3 (R^2^ = 0.00, p=0.90, **Figure 2F**). It should be emphasized that all participants were euthyroid. In order to assess the clinical relevance of our findings we stratified them into tertiles according to their free T4 levels (**Figure 3A**). Importantly, the mean TSH level in all tertiles was virtually identical (ANOVA p=0.89, **Figure 3B**).

In participants with a level of free T4 in the highest tertile (free T4 17.5 to 21.0 pM) mean CIT was approximately four-fold higher (10.2 ± 7.5% of EE_warm_) than in those in the lowest tertile (free T4 10.9 to 15.7 pM, mean CIT of 2.3 ± 8.7%, ANOVA p=0.0057, trend p=0.0027, **Figure 3C**).

### Multivariate Analysis of Factors Associated with Energy Expenditure and Cold Induced Thermogenesis

REE and CIT are known to be associated with several anthropometric factors and outdoor temperature (21, 22). Therefore, we performed multiple linear regression to analyze whether levels of TH were still predictive of EE_warm_, EE_cold_ or relative CIT.

EE_warm_ was significantly influenced by weight (p=0.023) and sex (p<0.0001) of participants (**Table 2A**). For EE_cold the_ outdoor temperature during the week before the measurement of REE was an additional significant predictor (p=0.0021) (**Table 2B**). In both models, levels of TH did not affect REE. However, levels of free T4 were significantly associated with CIT (p=0.020), as were outdoor temperature (p=0.0035), height (p=0.0005) and weight (p=0.0069) (**Table 2C**). We performed the same analysis also with the warm (mean temperature ≥ 15.0°C) *vs*. cold season and the meteorological season. In both models free T4 was a significant predictor of CIT (p=0.0085 and p=0.0088). CIT was inversely associated with the warm season as compared to the cold season (p=0.024). Using meteorological seasons as a factor and comparing to autumn, summer was inversely correlated with CIT (p=0.045), see **Supplementary Table 1**.

**Table 2:** Results of multiple linear regression.

A Energy Expenditure Warm					
Model: EEwarm ~ fT4 + Temp7d + Height + Weight + Sex					
	Value	Std. Error		t-value	p-value
**(Intercept)**	-1.22	0.29		-4.22	<0.0001
**fT4**	-0.10	0.08		-1.13	0.26
**Temp7d**	-0.11	0.08		-1.45	0.15
**Height**	0.16	0.11		1.47	0.15
**Weight**	0.27	0.11		2.32	**0.023**
**Sex**	1.39	0.32		4.37	**<0.0001**
Multiple R^2^: 0.60, Adjusted R^2^: 0.57					
**B Energy Expenditure Cold** **Model:** EEcold ~ fT4 + Temp7d + Height + Weight + Sex					
	**Value**	**Std. Error**		**t-value**	**p-value**
**(Intercept)**	-0.86	0.30		-2.90	0.0049
**fT4**	0.05	0.09		0.57	0.57
**Temp7d**	-0.25	0.08		-3.19	**0.0021**
**Height**	0.42	0.11		3.84	**0.0003**
**Weight**	-0.001	0.12		-0.01	0.99
**Sex**	0.99	0.33		3.00	**0.0037**
Multiple R^2^: 0.58, Adjusted R^2^: 0.55					
**C Cold Induced Thermogenesis** **Model:** CIT ~ fT4 + Temp7d + Height + Weight + Sex					
	**Value**	**Std. Error**		**t-value**	**p-value**
**(Intercept)**	0.27	0.37		0.72	0.48
**fT4**	0.26	0.11		2.37	**0.020**
**Temp7d**	-0.30	0.098		-3.02	**0.0035**
**Height**	0.50	0.14		3.64	**0.0005**
**Weight**	-0.41	0.15		-2.78	**0.0069**
**Sex**	-0.30	0.41		-0.74	0.46
Multiple R^2^: 0.34, Adjusted R^2^: 0.30					

Reported values are coefficients from multiple linear regression with CIT as dependent variable. All scalar factors were Z-scaled prior to analysis. Significant associations are indicated in bold.

### Association of Free T4 and Cold Induced Metabolic Activity of Brown Adipose Tissue

**Figure 4:**
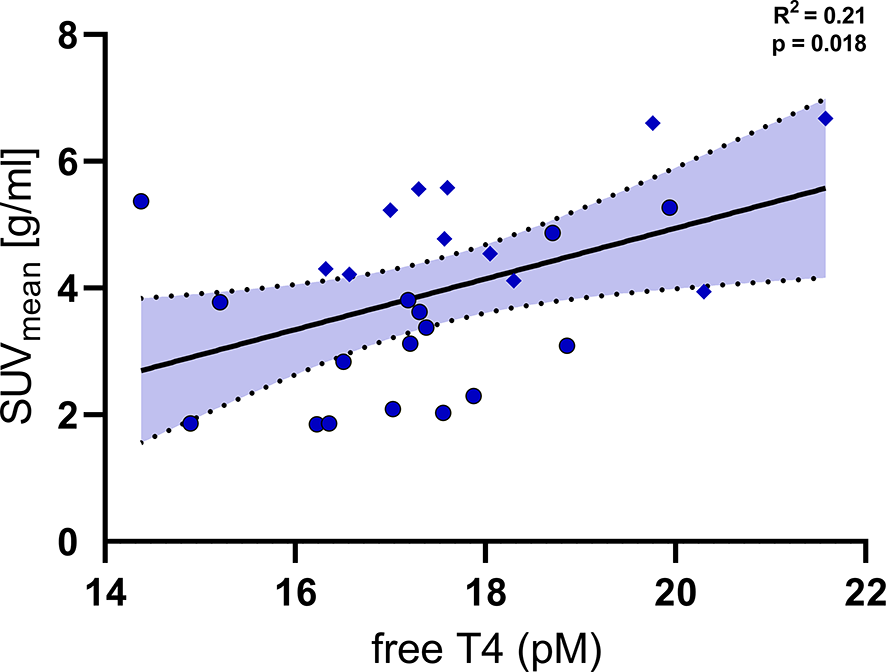
Levels of free T4 were significantly associated with the uptake of fluoro-deoxyglucose (SUV_mean_) into supraclavicular brown adipose tissue (R^2^ = 0.21, p=0.018). Diamonds: individual data points from trial 1 NCT03189511 (PET/MRI, BAT stimulation by Mirabegron and cold exposure), Circles: individual data points from trial 2 NCT03269747 (PET/CT, BAT stimulation by cold exposure).

In order to further delineate the effects of free T4 on cold induced metabolism we analysed PET/CT and PET/MR data which were available for a subset of the participants. ^18^F-FDG-PET/CT and –PET/MR had been performed after two hours of mild cold exposure and were compared to the levels of free T4 in a serum sample directly prior to BAT stimulation. SUV_mean_ of ^18^F-FDG in the supraclavicular adipose tissue was significantly associated with levels of free T4 (**Figure 4**, R^2^ = 0.21, p=0.018).

### Association of Thyroid Function Parameters and Outdoor Temperature

TH metabolism is increased during exposure to severe cold (23, 24). In the temperate climate of Basel, Switzerland, free T4 was weakly, albeit significantly associated with outdoor temperature (R^2^ = 0.06, p=0.037). This association persisted when weight and sex were added to the regression model. Free T3 and TSH were not related to outdoor temperature (R^2^ = 0.00, p=0.81, and R^2^ = 0.014, p=0.30, respectively, **Table 3**).

**Table 3:** Association of free T4 and outdoor temperature.

Association of Free T4 and Outdoor Temperature Model: fT4 ~ Temp7d + Weight + Sex				
	Value	Std. Error	t-value	p-value
**(Intercept)**	-1.36	0.36	-3.81	0.0003
**Temp7d**	-0.21	0.10	-2.03	**0.046**
**Weight**	-0.24	0.31	-1.84	0.07
**Sex**	1.56	0.39	3.98	**0.0002**
Multiple R^2^: 0.23, Adjusted R^2^: 0.19				

Reported values are coefficients from multiple linear regression with free T4 as dependent variable. All scalar factors were Z-scaled prior to analysis. Significant associations are indicated in bold.

Levels of thyroglobulin are an endogenous marker of thyroid hormone synthesis and have been shown to be substantially increased in very cold climates (23). In our cohort and a temperate climate zone we found no association of thyroglobulin to outdoor temperature (R^2^ = 0.03, p=0.14).

## Discussion

In this study, we demonstrate that levels of free T4 within the reference range in euthyroid, healthy individuals are significantly associated with CIT. This association persisted after multivariate correction for other parameters known to influence CIT. Further, we provide evidence that this effect may be caused at least partially by an influence on BAT activity.

In standard clinical practice, TSH is considered to be the most sensitive and cost-effective test of thyroid function. A value within the reference range is considered to be a robust marker of euthyroid hormone levels. All healthy volunteers participating in our study had normal serum TSH levels indicating normal thyroid function. However, measuring levels of free thyroxin (free T4) and free liothyronine (free T3) allows a more detailed overview of thyroid function. The population based reference ranges for free T4 and free T3 are relatively large (25–27) suggesting potential effects on metabolism. We therefore investigated whether these differences in levels of free T4 and free T3 affect human thermogenesis.

Previously, we and others were able to demonstrate that REE is reduced in hypothyroidism and that restoring euthyroidism normalizes REE in humans (17, 28). Here, higher levels of free T4 within the reference range did not lead to higher REE at room temperature in euthyroid individuals, which is in line with a recent study in a larger cohort (29). Interestingly, levels of free T3 were significantly associated with REE at room temperature. Of note, this association did not persist after multivariate correction. In a similar population as the one studied here, Roef et al. found a significant positive association of free T3 with BMI (30) which might explain this finding. As T4 is mainly converted to T3 intracellularly by DIO2 in brown adipocytes, the levels of T3 measured in serum do not necessarily reflect the levels within BAT (31).

In contrast, levels of free T4 but not free T3 were positively associated with CIT in our cohort. CIT reflects the increase in REE in response to cool environmental temperatures in order to maintain core body temperature. BAT is one of the main tissues to facilitate CIT and is a well-established target of TH action.

BAT expresses high amounts of deiodinase type 2 (DIO2) which locally converts T4 into T3 and which is induced by cold exposure (32, 33). These mechanisms are crucial for mitochondriogenesis and heat production (32). We assessed BAT activity by FDG-PET in a subset of our cohort. Indeed, we could demonstrate that higher levels of free T4 are significantly associated with a higher metabolic activity of BAT. In line with our data, circulating levels of free T4 within the reference range were significantly related to markers of white to brown adipocyte conversion in human subcutaneous adipose tissue (34).

Importantly, the main stimulus of CIT in adult humans appears to be cool environmental temperatures (21, 35, 36). Previously, we could demonstrate that in hypothyroid individuals the effect of environmental temperatures on CIT seems to be abrogated (17). Therefore, we would like to speculate that thyroid hormones facilitate the dynamic adaptations to cool temperatures also in humans. This notion is supported by findings in rats: thyroid ablation did not affect BAT function after animals had been acclimated to cool temperatures. However, if thyroid ablation was performed prior to chronic cold exposure animals exhibited blunted function of BAT (37).

Furthermore, BAT activity and CIT differ substantially between healthy individuals of comparable age and body composition. Importantly, a significant proportion of participants in our study reduced EE in response to cold exposure, i.e. had negative CIT. A recent study showed that individuals reacting with negative CIT to mild cold exposure do also reduce EE to a greater extent while fasting. The authors suggested that this phenomenon might therefore signify a “thrifty” phenotype (38).

In mice, strain specific differences in the ability to recruit BAT have been demonstrated and suggest strong genetic influences (39). In humans, genetic influences on BAT recruitment have also been demonstrated (40, 41). Still, the underlying genetic alterations, molecular mechanisms, and physiologic regulation have to be elucidated and might comprise transcription factors involved in adipocyte browning as well as thyroid hormone metabolism.

Our findings suggest, that slightly higher levels of free T4 allow for better adaptation to cold environments. An increase in CIT, e.g. by systematic mild cold exposure, has been demonstrated to improve insulin sensitivity (16) and reduce body fat mass (42). In line with these findings, large cohort studies in China and Belgium demonstrated favorable effects of higher levels of thyroxine within the reference range, in euthyroid individuals (11, 30), which might be facilitated in part by CIT.

Previously, exposure to severe cold in the polar regions has been demonstrated to increase TH metabolism in humans resulting in increased levels of thyroglobulin and iodine excretion. However, steady state levels of free T4 and free T3 were not increased or rather reduced in these studies (23, 24). In our cohort, levels of thyroglobulin were not associated with outdoor temperature or CIT, but free T4 was weakly associated with temperature. It should be noted that the outdoor temperatures in the cited papers were approximately 20 to 30°C lower than in our study and might thus lead to different adaptive effects. Systematic studies of TH levels in more temperate climate zones comprise relatively few participants (43). Some of these studies also indicate a seasonal influence on thyroid hormone levels (44). More recent analysis of very large datasets imply an inverse influence of outdoor temperature on TSH (45, 46).

Our study is limited by its observational nature and we can thus not prove causality. Strengths of our study comprise the relatively large cohort of participants and the thorough and prospective measurement of REE and CIT. Furthermore, we took into account the environmental temperatures which are a major factor influencing CIT. We could find evidence that BAT activity was positively associated with levels of free T4 in a subgroup of participants whom we investigated with FDG-PET.

In conclusion, we provide evidence for a link between TH and CIT in healthy individuals. Our findings suggest that differences in the level of TH even in euthyroid individuals affect metabolism significantly and should be studied in further detail.

## Data Availability Statement

The datasets used and/or analyzed during the current study are available from the corresponding author on reasonable request. The request will be judged by an independent committee at the Department of Clinical Research of the University Hospital Basel to ensure that legal obligations are fulfilled.

## Ethics Statement

The studies involving human participants were reviewed and approved by Ethikkommission Nordwest- und Zentralschweiz, Basel, CH. The patients/participants provided their written informed consent to participate in this study.

## Author Contributions

CIM and MJB: conceived the study, performed experiments, analyzed data, drafted the manuscript. JRS, RCL, JM, ASB, and MB: performed experiments, analyzed data. CW and IAB: conceived the study, analyzed data. MK: performed thyroid hormone measurements. All authors contributed to the article and approved the submitted version.

## Funding

The study was funded by grants from the Swiss National Science Foundation (grant no. PZ00P3_167823), the Bangerter-Rhyner Foundation, Basel, and the Nora van der Meuuwen-Häfliger Foundation, Basel, to MJB. RL received a stipend from the Goldschmidt-Jacobson Foundation, Basel.

## Conflict of Interest

The authors declare that the research was conducted in the absence of any commercial or financial relationships that could be construed as a potential conflict of interest.

## Abbreviations

AMPK, Adenosine Monophosphate-Activated Protein Kinase; BAT, Brown Adipose Tissue; BMI, Body Mass Index; CIT, Cold Induced Thermogenesis; DIO 2, Deiodinase 2; EE, Energy Expenditure; FDG, Fluordesoxyglucose; fT3, Free Triiodothyronine; fT4, Free Thyroxine; TH, Thyroid Hormone; TSH, Thyroid Stimulating Hormone; UCP1, Uncoupling Protein 1; REE, Resting Energy Expenditure.
