# Supplementary material for: Free Thyroxine Levels are Associated with Cold Induced Thermogenesis in Healthy Euthyroid Individuals
Source: Front Endocrinol (Lausanne). 2021 Jun 14;12:666595. doi: 10.3389/fendo.2021.666595 (PMC8236885; doi:10.3389/fendo.2021.666595)
Supplement: Supplementary file 1 [file Table_1.docx]

| **Cold Induced Thermogenesis: Influence of Warm Season**  **Model:**  CIT ~ fT4 + Warm_Season + Height + Weight + Sex | | | | |
| --- | --- | --- | --- | --- |
|  | **Value** | **Std. Error** | **t-value** | **p-value** |
| **(Intercept)** | 0.50 | 0.39 | 1.30 | 0.20 |
| **fT4** | 0.30 | 0.11 | 2.70 | **0.0085** |
| **Warm Season** | -0.45 | 0.20 | -2.31 | **0.024** |
| **Height** | 0.49 | 0.14 | 3.51 | **0.00078** |
| **Weight** | -0.37 | 0.15 | -2.47 | **0.016** |
| **Sex** | -0.33 | 0.42 | -0.80 | 0.43 |
| Multiple R^2^: 0.31, Adjusted R^2^: 0.26 | | | | |

Supplementary Table 1:

**A**

Warm Season: Average daily temperature during seven preceding days ≥ 15°C, n=37 vs. cold season average daily temperature < 15°C n=42.

**B**

| **Cold Induced Thermogenesis: Influence of Meteorological Season**  **Model:**  CIT ~ fT4 + Met_Season + Height + Weight + Sex | | | | |
| --- | --- | --- | --- | --- |
|  | **Value** | **Std. Error** | **t-value** | **p-value** |
| **(Intercept)** | 0.45 | 0.44 | 1.01 | 0.32 |
| **fT4** | 0.31 | 0.11 | 2.69 | **0.0088** |
| **Season_Spring** | 0.039 | 0.29 | 0.13 | 0.89 |
| **Season_Summer** | -0.51 | 0.25 | -2.04 | **0.045** |
| **Season_Winter** | 0.25 | 0.28 | 0.89 | 0.37 |
| **Height** | 0.53 | 0.14 | 3.78 | **0.0003** |
| **Weight** | -0.38 | 0.15 | -2.49 | **0.015** |
| **Sex** | -0.41 | 0.43 | -0.95 | 0.34 |
| Multiple R^2^: 0.34, Adjusted R^2^: 0.27 | | | | |

Seasons:

Winter Dec 1^st^ to Feb 28^th^

Spring Mar 1^st^ to May 31^st^

Summer Jun 1^st^ to Aug 31^st^

Autumn Sep 1^st^ to Nov 30^th^ (comparator)
